# Commentary on: Combination of Metabolic Intervention and T Cell Therapy Enhances Solid Tumor Immunotherapy

**DOI:** 10.20900/immunometab20210016

**Published:** 2021

**Authors:** Anthos Christofides, Natalia M. Tijaro-Ovalle, Vassiliki A. Boussiotis

**Affiliations:** 1 Division of Hematology-Oncology, Beth Israel Deaconess Medical Center, Harvard Medical School, Boston, MA 02215, USA; 2 Department of Medicine, Beth Israel Deaconess Medical Center, Harvard Medical School, Boston, MA 02215, USA; 3 Cancer Center, Beth Israel Deaconess Medical Center, Harvard Medical School, Boston, MA 02215, USA; 4 Yale School of Medicine, New Haven, CT 06510, USA

**Keywords:** T cell activation, CAR T cells, cancer immunotherapy, cholesterol

## Abstract

Metabolism is a common cellular feature. Cancer creates a suppressive microenvironment resulting in inactivation of antigen-specific T cells by metabolic reprogramming. Development of approaches that enhance and sustain physiologic properties of T cell metabolism to prevent T cell inactivation and promote effector function in the tumor microenvironment is an urgent need for improvement of cell-based cancer immunotherapies.

T cells are central regulators of anti-tumor immunity [1]. Cytolytic CD8^+^ T lymphocytes (CTL) can mediate direct cytotoxic effects on tumor cells, whereas helper CD4^+^ T cells provide help for CTL function but also mediate direct cytotoxic activity. Due to these well-established anti-tumor properties, T cell-based immunotherapies, particularly chimeric antigen receptor (CAR) T cells, have extended our armories in the battle against cancer and, together with checkpoint inhibitor blockade, have revolutionized cancer immunotherapy [2]. Although considerable success has been achieved in the treatment of hematologic malignancies [3,4], the success of T cell immunotherapies in the context of solid tumors has been limited [5–7]. Various factors account for this therapeutic dichotomy, the most critical of which include the paucity of targetable tumor-associated antigen(s) and the limited functionality of the adoptively transferred therapeutic T cells in the tumor microenvironment (TME) [8,9].

Metabolism, is a key mechanism that shapes the properties of the TME. Cancer creates a hostile metabolic microenvironment by various mechanisms including nutrient deprivation, metabolic competition, hypoxia, lactate production and oxidative stress [10,11]. These conditions have a significant impact in the function of T cells residing in proximity to cancer [12,13]. Metabolic reprogramming of T cells is imperative for their differentiation and function. This process occurs in a well-coordinated and temporally defined manner and is mandatory for T cell activation and differentiation. While naïve and memory T cells rely mostly on oxidative metabolism, where lipids have a central role, glycolysis has an indispensable role for generation and expansion of T effector cells [14]. In addition to being involved in T cell metabolism, lipids have a distinct role in antigen-mediated signal initiation. Activation of T cell signaling requires TCR clustering in the lipid rafts and formation of immune synapse, processes that rely on intramembranous cholesterol and are highly dependent on the density and availability of lipids at the plasma membrane [15]. T cells generate such membrane lipids during anabolic metabolism initiated by antigen-mediated activation [16]. In the TME, cancer-mediated coordinated metabolic switches, modulate T cell metabolic properties and cellular activities and compromise lipid metabolism [17]. As a consequence, T cells lose their ability to synthesize lipids that regulate clustering of signalosomes and transmission of TCR-mediated signals and become unable to respond to stimulation by cancer-associated antigens. This is a key mechanism of cancer-mediated immune escape leading to cancer progression.

A recent study attempted to target this specific step of T cell activation by employing a novel approach to enhance initiation of T cell signaling in order to overcome the detrimental challenges of the TME [18]. Because TCR clustering and stabilization of the immunological synapse rely on intramembranous cholesterol, inhibition of cholesterol esterification enzymes, which increase the levels of membrane cholesterol, improve T cell activation and effector function [19]. To recapitulate this process the authors engineered the metabolism modulating drug Avasimibe (Ava), an inhibitor of acetyl-CoA acetyltransferase 1 (ACAT1) [20], to sustain its presence at the plasma membrane. After implementation of a cell surface anchor-engineering technology facilitated by the insertion of tetrazine (Tre) groups in the plasma membrane [21], liposomal Ava containing bicyclo [6.1.0] nonyne (BCN) was successfully retained at the T cell surface (Figure 1A). This approach inhibited cholesterol esterification, and enhanced the fraction of cholesterol present at the cell membrane. Notably, cell surface anchored Ava (T-Tre/BCN-Lipo-Ava) did not alter T cell viability, survival, activation, basal metabolism or chemotaxis. Instead, T-Tre/BCN-Lipo-Ava increased plasma membrane cholesterol, amplified the clustering of TCRs, and resulted in the formation of enhanced and stabilized immunological synapse. This led to more efficient TCR downstream signaling and production of IL-2, IFNγ and TNFα, ultimately elevating T cell anti-tumor activity (Figure 1B). More importantly, TCR-transgenic and CAR-T cells engineered to carry Tre/BCN-Lipo-Ava exhibited greater anti-tumor capacity than unmodified T cells. This was demonstrated by their higher tumor cell-killing capacity when co-cultured with melanoma cells in vitro, and delayed tumor progression in vivo after adaptive transfer into melanoma- or glioblastoma-bearing mice [18].

This study provides evidence for the first time that this cell-surface anchor-engineering approach provides the opportunity to use high concentrations of metabolism-modulating drugs to target specifically and directly antigen-specific T cells prior to adoptive transfer. This approach minimizes the non-specific effects that might arise from systemic administration of a drug with the purpose and hope to target T cells of the TME. This approach provides a sustained metabolism-modifying impact that overcomes the detrimental implications of the TME on the specific module of T cell metabolism that governs TCR signal transduction. Importantly, this seems to have a long-lasting impact on T cell function.

The recent success in anchoring an active drug at the cell plasma membrane opens new avenues in therapeutic intervention at a cell-specific manner. This approach is promising particularly in the context of cell-based therapies, where ex vivo or in vitro modification prior to adoptive transfer is feasible. Of note, although in vitro metabolic modulation of T cells before adaptive transfer has been previously proposed and tested, this approach is limited by the metabolically hostile TME that is capable of alternating the intrinsic metabolic state of infiltrating T cells. Anchoring a metabolism-modulating drug in tumor-specific T cells prior to adoptive transfer might overcome this limitation and allow therapeutic exploitation of metabolism for improvement of T cell function in cancer therapy.

Several challenges and questions remain to be answered. For example: how stable is the maintenance of a membrane-anchored engineered compound? Do engineered T cells undergo unimpeded divisions after antigen encounter despite the modification? Do daughter cells preserve the engineered compound on their cell surface, and at what levels? Can engineered T cells undergo differentiation to T memory cells after exposure to tumor-associated antigens in the TME and survive beyond effector phase to provide immune surveillance that prevents cancer relapse? Can such modifications be implemented for therapeutic alteration of intracellular molecules such as enzymes or transcription factors that imprint distinct metabolic and functional fates in T cells? Despite the many unanswered questions, the new technology provides hope for a new era in the generation of engineered antigen-specific T cells for improvement of cell-based immunotherapies in cancer.

## Figures and Tables

**Figure 1.**
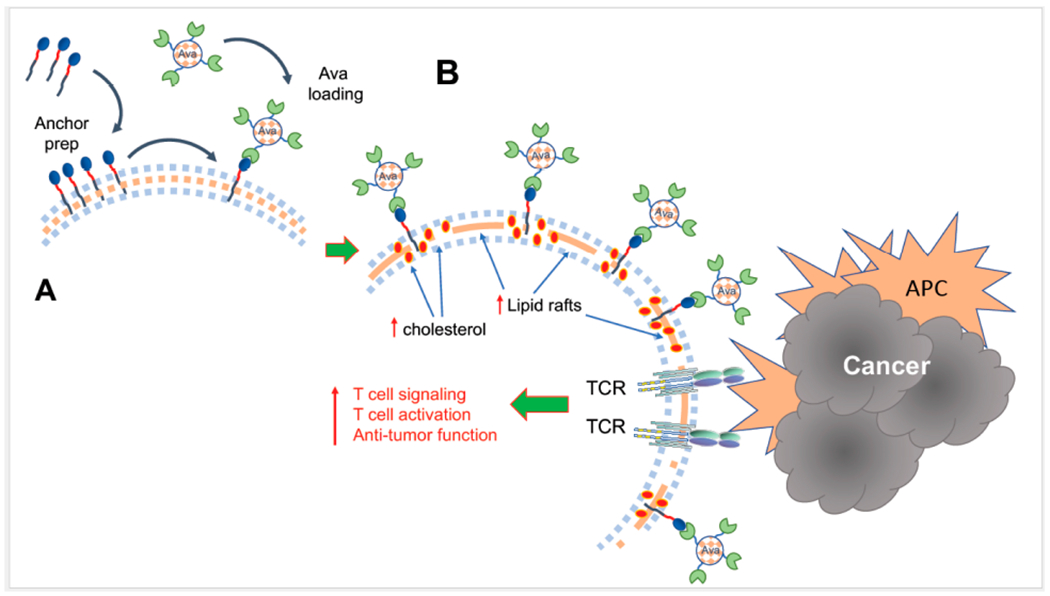
(**A**) Implementation of a cell surface anchor-engineering technology facilitated by the insertion of tetrazine (Tre) groups in the plasma membrane allowed capture of liposomal Ava containing bicyclo [6.1.0] nonyne (BCN) at the T cell surface. (**B**) This approach increased the fraction of cholesterol present at the cell membrane, leading to enhanced formation of lipid rafts, TCR aggregation and amplification of TCR signaling after engagement by tumor antigens presented by antigen presenting cells (APC), resulting in augmented T cell activation and antitumor function.
